# FAMILY DEMENTIA CAREGIVER RECRUITMENT STRATEGIES IN LONG-TERM CARE FACILITIES

**DOI:** 10.1093/geroni/igz038.3395

**Published:** 2019-11-08

**Authors:** Olimpia Paun, Ben R Inventor, Louis Fogg, Hugh Vondracek, Ilse Salinas

**Affiliations:** Rush University College of Nursing, Chicago, Illinois, United States

## Abstract

Recruitment of dementia caregivers whose family members reside in long-term care facilities (LTCFs) poses unique challenges as it traditionally relies on the assistance of facility administrators. The purpose of this presentation is to examine and evaluate new recruitment strategies to determine their effectiveness in an ongoing Stage I randomized clinical trial testing the effects of a Chronic Grief Management Intervention, Video-streamed (CGMI-V) on caregivers’ mental health (grief, depression, anxiety) and facility-related outcomes (conflict with staff, satisfaction with care). A total of 144 caregivers will be randomly assigned to the CGMI-V or to the minimal treatment conditions. The initial recruitment plan was to build on already-established relationships with more than 35 LTCFs that helped recruit in a previous study. The usual approach was to offer written materials and onsite presentations about the study to facility staff and to dementia family caregivers of facility residents. Within the first six months, recruitment efforts yielded less than a dozen participants, thus we had to refine our approach. Revised recruitment strategies included the adoption of resources from the National Institute on Aging’s ADORE (Alzheimer’s and Dementia Outreach, Recruitment, and Engagement) and ROAR (Recruiting Older Adults into Research) platforms. This new approach included online study advertising on NIH and Alzheimer’s Association research study repositories and advertising on parent institution’s on-hold messaging system. Adoption of these new strategies is yielding an increase in participant screening and enrollment. Results are pending.

